# Plant-Derived Extracellular Vesicles as Therapeutic Nanocarriers

**DOI:** 10.3390/ijms23010191

**Published:** 2021-12-24

**Authors:** Theodora Karamanidou, Alexander Tsouknidas

**Affiliations:** 1 Laboratory for Biomaterials and Computational Mechanics, Department of Mechanical Engineering, University of Western Macedonia, 50100 Kozani, Greece; atsouknidas@uowm.gr; 2 Graduate Program in Biomedical Engineering, Department of Mechanical Engineering, University of Western Macedonia, 50100 Kozani, Greece

**Keywords:** nanovesicles, exosome-like vesicles, intercellular communication, cancer treatment

## Abstract

Mammalian exosomes have emerged as a promising class of functional materials, inspiring novel applications as therapeutic vehicles and nutraceutical compounds. Despite this, their immunogenicity has been an issue of controversy within the scientific community. Although, exosome-like vesicles, innately formed in plants and inherent to eukaryotic cell-derived vesicles, could soothe most of the concerns, they are notably underutilized as therapeutic modalities. This review highlights all efforts published so far, on the use of plant-derived extracellular vesicles (EVs) as therapeutic delivery systems. A summary of the physicochemical characteristics of plant-derived EVs is provided along with their main biological composition and in vitro/in vivo evidence of their therapeutic efficacy provided where available. Despite only a hand full of clinical trials being underway, concerning these vesicles, they arguably possess significant potential as nanodelivery systems of natural origin.

## 1. Introduction

Exosomes are intraluminal vesicles, bearing a protein and nucleic acid cargo. Released during exocytosis, they function as extracellular vesicles (EVs) that participate in the intercellular communication of various physiological and pathological processes. Despite being sized in the nanodomain, they are considered natural/biodegradable media. Since their first discovery in 1983 in mammalian cells [1,2], extensive research has been performed in various scientific sectors, including, among others, drug delivery systems, nutrition, clinical diagnostics and therapeutics.

**Figure 1:**
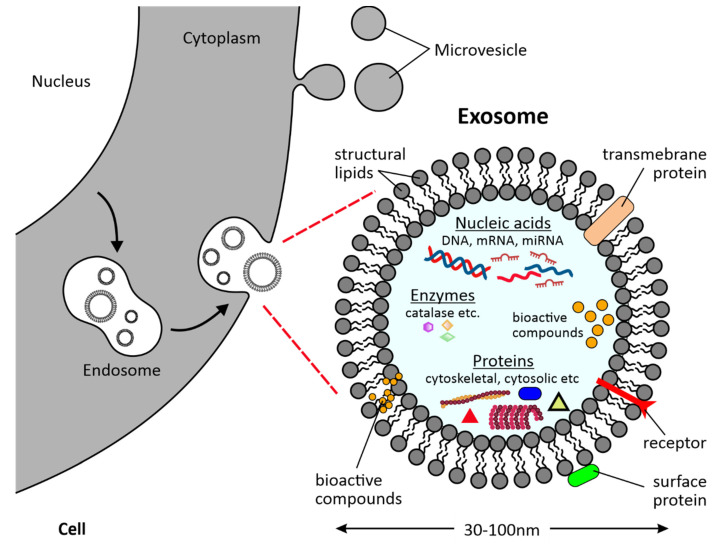
Structure of an exosome released during exocytosis and used as a delivery vesicle for bioactive compounds (which can be added after their isolation).

Despite the molecular signature of most exosomes, exhibiting the similar structural characteristics, (e.g., nucleic acids, proteins, carbohydrates and lipid composition), their uptake and signaling capacity is largely dependent on their origin or state and may even vary based on their isolation path [3]. Recent studies have shown the potential of exosomes as delivery vesicles for bioactive compounds [4], that may either be part of their cargo, or lipid structure, see Figure 1.

Extracellular vesicles, derived from plants, have a similar structural composition to mammalian exosomes and could address the growing demand for the intra-nutrial delivery of innate bioactive compounds [5,6,7], or their utilization as therapeutic delivery systems [7,8,9,10,11,12,13], based on their ability to withstand the activity of digestive enzymes [7,9,14].

Wang et al. (2015) recently advocated that a nutrient uptake of plant-derived nanovesicles has multiple functions which could result in health benefits, while also bearing great potential for the efficient delivery of therapeutics, without eliciting inflammatory responses [15].

These health-related aspects of plant-derived extracellular vesicles are reviewed, along with an overview of all related EVs found in current literature.

## 2. Plant-Derived Extracellular Vesicles Classified by FAO Group

During recent years, EVs have been isolated from various plant species and different sections thereof (i.e., fruits, roots, seeds, leaves), using standardized isolation and purification techniques e.g., ultracentrifugation [16].

Table 1 summarizes the current literature on EVs, extracted from plants, classified according to the Food and Agriculture Organization (FAO) group categories. The most widely used category is fruits (Group 8) followed by vegetables (Group 7) and spices (Group 10). Within Group 8, grapes [7,8,9,17,18] and grapefruits [7,9,12,15,17,19,20] are the most well studied fruits for the isolation and therapeutic efficacy of EVs, followed by ginger, an edible root, in the category of spices, that presents high drug-delivery efficiency and significant therapeutic potential [9,10,11,12,14,20,21]. As can be observed in Table 1, many studies have been conducted, using common, edible plants, such as oranges, lemons and tomatoes etc. [5,7,20,22] and other less usual and more demanding ones, with respect to sample handling, such as coconut, sunflower seeds and cactuses [12,20,23,24].

The most documented techniques for the isolation/purification of plant-derived nanovesicles are: differential centrifugation/ultracentrifugation, density gradient centrifugation (e.g., using sucrose) and filtration (Table 1). However, due to the numerous limitations of these techniques, such as complexity, low yield, long run time intervals and impurities, scientists have suggested the use of alternative and more innovative strategies, to optimize the extraction procedure for EVs, e.g., centrifuge-based ultrafiltration, microfluidics and nanoplasmon-enhanced scattering [16]. Grouping these techniques into two main categories, based on the initial steps of EV isolation, the majority of studies (approximately 61%) employ density gradient centrifugation, whereas the remaining 39% are based on differential centrifugation/filtration/ultracentrifugation (see Table 1 for details).

## 3. Physicochemical Characterization of Plant-Derived EVs

Plant-derived nanovesicles have shown significant therapeutic potential, based on their biological cargo [8,17]. EVs can be used either as they are, transferring their own biological cargo (e.g., nucleic acids, bioactive lipids, cell-surface proteins), or as delivery systems for other active ingredients, due to their biocompatibility and adjustable nature. Table 2 summarizes the primary physicochemical characteristics (i.e., size, morphology, z-potential) of plant-derived EVs with, or without the incorporation of therapeutic biomolecules.

As illustrated in Table 2, EVs can acquire a particle size between 10 nm–1 μm, with a spherical, oval or cup-shaped morphology and a negative surface charge, all dependent on plant species and extraction procedure.

In vitro enzymatic digestion studies have shown that EVs are stable in gastric and/or intestinal simulating fluids, with respect to their physicochemical features (i.e., size, size distribution, surface charge), indicating their potential as nutritional carriers if fruits and/or vegetables are included in someone’s diet as well as oral drug delivery systems [7,9,25]. The latter aspect is further established by several experimental studies, where plant-derived EVs are administered orally for the treatment of various diseases, such as colitis, bowel and liver diseases, and Alzheimer’s disease [7,8,9,11,12,13,19]. On the other hand, studies by Zhuang et al. have shown that the physicochemical characteristics of ginger-derived EVs, such as size and surface charge, can be significantly affected after their incubation in simulated stomach solution (pH 2) or simulated small intestine medium (pH 6.5) [19]. However, this drawback could be easily overcome, using enteric coated tablets or capsules that bypass the stomach’s harsh environment and release their therapeutic cargo at the site of interest.

Another critical aspect of lipid particles, such as EVs, is the evaluation of storage stability and the selection of the most suitable storage conditions, to avoid undesirable phenomena (e.g., aggregation) that may have a negative impact on an EVs potential. Re-assembled ginger-derived nanovesicles (GiNVs) exhibit significant stability based on size distribution and z-potential analysis, after incubation at 4 °C for 25 days [9,24]. However, EVs should be stored in deep-freeze conditions (−80 °C) [5,12,20,23,26] to retain the biological molecules’ integrity (e.g., mRNA), since exposure to erratic temperatures can lead to undesirable degradation/damage, due to their fragile nature. In addition, EVs extracted from dried leaves of various plants (e.g., tobacco) appear to be stable as delivery vehicles, despite the additional drying process which can cause severe osmotic stress and possible damage to the EVs’ structure [27].

## 4. Molecular Composition of Plant-Derived EVs

Plant-derived nanovesicles function as extracellular messengers for intercellular communication, delivering biological molecules, such as lipids, proteins and nucleic acids. Therefore, the evaluation of EVs’ biological cargo, which depends, among other things, on their origin, is of significance and the presence/extent of this cargo, is considered an important metric for the successful isolation of these nanocarriers [8,9].

The function of EVs and their capacity to be taken up by cells, are both related to their lipid composition and lipidic structural assembling [11,17,28]. The lipidomic profile of plant-derived EVs is illustrated in Table 3. Phosphatidic acid (PA), which has recently emerged as an important lipid agent in intercellular communication, with key characteristics in drug delivery, is interestingly one of the most prevalent lipidic molecules found in plant-derived EVs [8,10,11,14,21,24,28]. Sundaram et al. have studied the effect of GiNVs on the periodontal pathogen *Porphyromonas gingivalis* and the importance of PA in the uptake of EVs by bacteria. This study group demonstrated that PA ratio highly affects the interaction of EVs with hemin-binding protein 35, thus regulating the pathogenic activity of recipient bacteria, *P. gingivali* [21]. In another study [29], the uptake of GiNVs by *Lactobacillus rhamnosus* was evaluated and again, found to be PA dependent. Teng et al. (2018) demonstrated that grapefruit-derived nanovesicles (GrfNVs) enriched with lipid phosphatidylcholine (PC) were preferentially taken up by Ruminococcaceae, indicating that lipid composition plays a key role in cellular uptake mechanisms. The presence of specific lipids has also been identified as an important factor in tissue targeting [29], suggesting that PC enhances the migration of EVs from intestine to liver, while PA can increase the accumulation and duration time of EVs in the intestine as well [29]. Wang et al. showed that PC and phosphatidylethanolamine (PE) are the prevalent lipids in GrfNVs, providing enhanced antioxidant, anti-inflammatory and anticolitic action [7]. Among other lipid molecules, plant-derived EVs frequently comprise of digalactosyldiacylglycerol (DGDG) and monogalactosyldiacyglycerol (MGDG), which are important glycolipids, responsible for several cellular functions in both health and disease (Table 3) and are frequently used as stabilizing agents in various lipid particles, such as liposomes [28]. Another important aspect, regarding EVs’ lipid composition, is the ability to reassemble extracted lipids into artificial nanovesicles, using conventional liposome synthesis techniques (e.g., homogenization, thin-film hydration). Several studies have successfully studied reassembled EVs, with optimal characteristics, as efficient therapeutic delivery vectors [7,11,19].

With respect to protein molecules, the innate protein cargo of EVs is transferred to the recipient cells, inducing various cellular functions. Thus, the proteomic profile of EVs is an important criterion in demonstrating the existence of exosome-like vesicles and to evaluate their therapeutic potential. Protein composition can be determined, using mass spectrometry and liquid chromatography; and protein concentration can be evaluated, using various protein quantification assay kits (e.g., Bio-rad, BCA, ToPA Bradford) [5,8,9,10,18,22,24,30,31]. Nevertheless, the proteomic profile of EVs represents an important research field of its own and thus cannot be covered within this review as it deserves a more detailed and focused analysis.

Over the years, many questions have been raised about whether the RNAs of plant-derived EVs mediate cell-cell communication and ergo, contribute to cell functions that are associated with health or diseases. Plant-derived EVs contain high amounts of microRNAs (miRNAs), a class of small noncoding RNAs, which are essential in various physiological and pathological processes, such as cell proliferation, cell death, metabolism and immune responses [20,21,22,23]. Studies by Teng et al. [29] examine four different plant-derived EVs (carrot, garlic, grapes and ginger) with reference to RNA presence, using gel electrophoresis and sequencing analysis. The results indicated that the examined EVs carry small-sized RNAs and miRNAs that can modulate microbiome composition and inhibit inflammatory diseases, such as colitis, suggesting that dietary-derived EVs, obtained through nutrition, can impact the regulation of gut microbiota. Many scientists have also correlated the existence of specific miRNA molecules (mi168a [32], miR159 [33]) with food intake from edible plants, suggesting that exogenous plant-derived miRNAs, acquired orally through nutrition, can participate in the regulation of mammalian gene expression. In an extensive study of eleven fruits and vegetables by Xiao et al., the existence of various miRNA species were identified that regulate the expression of cancer-related genes and inflammatory cytokines (in vitro results) [20]. Zhao et al. investigated the differences between mature and immature coconut water with respect to miRNA content, determining higher values in the latter by real-time PCR, thus predicting their potential effect on the human transcriptome [23]. In conclusion, dietary extracellular miRNA can be delivered and absorbed through the gastrointestinal tract, affecting gene expression. Consequently, the proteomic profile may be an important aspect of EVs but not solely responsible for their cell-cell communication capacity. As a result, the nucleic acid profiles are essential in the characterization of exosome-like vesicles and should thus be an important part of standard analysis by scientific studies [5,8,9,18,23,30].

**Figure 2:**
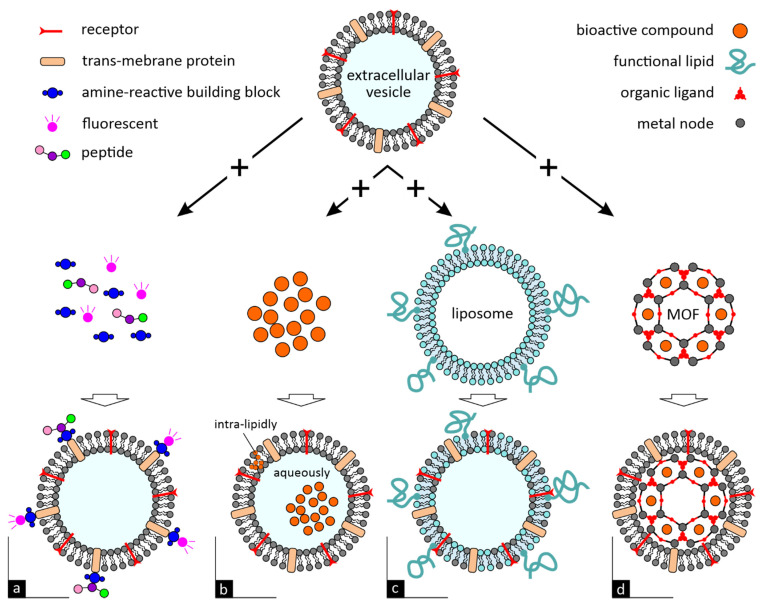
Typical modification of extracellular vesicles employed as therapeutic nanocarriers, of endemic and/or loaded cargo (**a**) surface modification, (**b**) encapsulation, (**c**) membrane fusion and (**d**) exosome-coating of nanoparticles (here shown for metal-organic frameworks, loaded with bioactive compounds).

In addition to the aforementioned biological molecules, EVs can also deliver innate active compounds to human cells. Baldini et al. have showed that EVs extracted from lemons (LEVs) carry micronutrients, such as Vitamin C and citrate, exerting antioxidant effects in human cells. This could be attributed to the cellular uptake of LEVs and the direct delivery and preservation of their unstable active components (ascorbic acid) [5]. Studies have detected amounts of naringin (a flavonoid) and its metabolite, functional naringenin, in GrfNVs, indicating the pharmacological potential of these EVs, due to the anti-inflammatory, antioxidant, anticolitic and anticancer effect of naringenin [7]. GiNVs were found to carry 6-gingerol and 6-shogaol, two anticancer, antioxidant and anti-inflammatory bioactive compounds [10,14]. Zhuang et al. have further identified that shogaol compound can have a protective effect in the development of liver-related diseases due to its action in the induction of nuclear factor erythroid 2-related factor 2 (Nrf2), a modulator of several cellular processes [10]. Finally, broccoli-derived EVs, containing sulforaphane, an active component found in some vegetables, contributed in the prevention of colitis in mice [13]. All the aforementioned innate active molecules can be transferred by delivery mechanism (a) or (b) as illustrated in Figure 2.

## 5. In Vitro and In Vivo Evidence of Therapeutic Efficacy of Plant-Derived EVs

In the present section, key results of in vitro and in vivo studies of plant-derived EVs are summarized for health- and disease-related applications. Sundaram et al. investigated the potential of GiNVs as therapeutic agents to ameliorate or prevent chronic periodontitis. The in vitro and in vivo results suggested that GiNVs can target *P. gingivalis*, a key pathogen in the development of oral disease periodontitis, and minimize alveolar bone loss and inflammation [21]. In other research, GiNVs targeted intestinal epithelial cells and macrophages, prevented various inflammatory bowel diseases, such as chronic and acute colitis or colitis-related cancer, and promoted intestinal mucosa healing [25]. Similarly, GiNVs, conjugated with folic acid (mechanism (a) as illustrated in Figure 2) and loaded with doxorubicin (a chemotherapy drug, mechanism (b) as illustrated in Figure 2), were effectively taken up by colon cancer cells successfully inhibiting colon tumor growth [14]. A novel siRNA-CD98 delivery vehicle, synthesized by Zhang et al. using GiNVs, efficiently reduced the expression of CD98, a glycoprotein related to colitis and colitis-associated cancer [11]. In another study, concerning the inflammation of the colon, gastrointestinal microbiota was found to selectively uptake GiNVs, improving mouse colitis by changing microbiome compositions and host physiology [29]. Zhuang et al. (2015) introduced a mouse model to determine the effectiveness of GiNVs in liver protection and their results demonstrated that orally administered GiNVs can provide liver protection against various diseases, such as alcohol-related liver damage [10]. Chen et al. (2019) examined the effectiveness of nine plant-derived EVs (vegetables, fruits, spices) on NLRP3 inflammasome inhibition, which is a key regulator of the innate immune system associated with the pathogenesis of multiple diseases (e.g., of inflammatory-, neurodegenerative-, metabolic-nature). Among the nine EVs, GiNVs presented enhanced targeting potential in primary macrophages, that restrained NLRP3 activation, indicating the potential for new therapeutic modalities in the treatment of complex diseases [12].

The potential of GrfNVs has repeatedly been investigated by many scientific groups. The targeting specificity and the efficient delivery of paclitaxel (a chemotherapeutic drug, mechanism (b) as illustrated in Figure 2), using GrfNVs conjugated with folic acid (mechanism (a) as illustrated in Figure 2), were investigated in two tumor animal models in which efficient inhibition of tumor growth was attained [15]. GrfNVs loaded with miR-18a (mechanism (b) as illustrated in Figure 2), a tumor suppressor, were found to induce inhibition of liver metastasis, by regulating macrophage M1 populations [34]. The results of Wang et al. (2014) showed that GrfNVs can work as immune modulators and attenuate dextran-sulfate sodium (DSS)-induced mouse colitis. In the same research, GrfNVs loaded with methotrexate (an anti-inflammatory drug, mechanism (a) as illustrated in Figure 2) minimized the drug’s toxicity with respect to free drug formulation, while also enhancing the therapeutic effect on DSS-induced mouse colitis [7]. In another study, GrfNVs were surface modified with membranes of activated leukocytes (mechanism (a) as illustrated in Figure 2), to increase the targeting potential and efficient delivery of either doxorubicin or curcumin (mechanism (b) as illustrated in Figure 2) to the site of interest. These vesicles presented effective delivery of drugs to the inflammatory site, leading to inhibition of colon and breast tumor growth, along with a therapeutic effect on DSS-induced colitis [15]. Zhuang et al. (2016) investigated the delivery of the miR17 molecule by surface modified GrfNVs (mechanism (a) as illustrated in Figure 2), for the inhibition of brain tumor growth after intranasal administration in mice. GrfNVs coated with either folic acid (FA) or both FA and polyethyleneimine, presented enhanced target potential in brain tumor cells and inhibition of tumor growth, leading to a new generation of noninvasive treatments via an intranasal delivery route for brain-related diseases [19].

Recent studies have also shown that grape-derived nanovesicles (GrpNVs) can have a protective and/or therapeutic effect on intestinal tissue renewal and DSS-induced colitis after their oral administration in mice [8]. In vitro studies of LEVs, showed that these vehicles can be selectively taken up by mesenchymal stem cells (MSC), modulating their differentiation towards osteogenic lineage, while also providing significant protection against oxidative stress [5]. In another study, LEVs were examined (in vitro) with respect to their antineoplastic activity on several tumor cell lines, showing significant antineoplastic potential, while the in vivo xenograft model of chronic myeloid leukemia exhibited reduced tumor growth [22]. Mu et al. (2014) extensively investigated the role of four plant-derived EVs (carrots, grapes, grapefruits and ginger) on intercellular communication. Both, the in vitro and in vivo results suggested that plant-derived EVs can modulate intestinal homeostasis by mediating various cellular mechanisms [9]. Finally, broccoli-derived EVs could selectively be taken up by dendritic cells and maintain intestinal immune homeostasis, thus preventing or even treating intestinal-associated inflammatory diseases, such as colitis [13].

## 6. Clinical Trials

Several clinical trials have been conducted and completed over the years, including exosomes from mammalian cells [35]. Only recently, did plant-derived EVs enter the recruitment processes for clinical trials. Table 4 summarizes the clinical trials that are currently underway, either in a recruiting or a beginning stage (Phase I Clinical Trial).

A clinical trial at Phase I, is currently being conducted to evaluate the delivery potential of plant-derived EVs conjugated with curcumin, an anti-inflammatory agent, in both normal and colon cancer tissue (ClinicalTrials.gov Identifier: NCT01294072) [36]. In addition, clinical trials have been developed to study the oral administration of GrpNVs to abrogate oral mucositis related with chemotherapy treatment of neck and head cancer (ClinicalTrials.gov Identifier: NCT01668849) [37]. Finally, the ability of GiNVs and aloe-derived EVs to mitigate insulin resistance and chronic inflammation in polycystic ovary syndrome (POS) patients will also be evaluated in clinical trials with reference number NCT03493984 after the completion of the recruiting process (ClinicalTrials.gov Identifier: NCT03493984) [38].

## 7. Conclusions

Cell-to-cell communication is predominantly based on chemical messengers. Plant derived extracellular vesicles can mediate targeted intracellular communication, rendering them an attractive, yet underutilized therapeutic modality. EVs can reach distant organs, attaching to cellular membranes through receptor–ligand interactions, releasing their functional cargo. The physicochemical composition of these nanocarriers, allows them to deliver their cargo directly to the cytoplasm of the recipient cell, avoiding the endosomal pathway and lysosomal degradation.

The significant advancements of EVs, associated with their potential as drug delivery systems, can be categorized into four basic delivery/modification techniques, as illustrated in Figure 2.

- Surface modifications to improve their targeting ability or monitor their delivery [39].
- Encapsulation of biomolecules, either in their lipid structure, or directly loaded into their aqueous core [7,15,17].
- Fusion with liposomes, to elicit a lower immune response and increase its colloidal stability [40].
- Exosome-coated metal–organic framework nanoparticles [41], that facilitate targeted delivery without premature leakage.

As outlined in the present review, plant-derived EVs can deliver active biomolecules via mechanism (a) and/or (b), while delivery of biomolecules/nanoparticles by techniques (c) and (d) have already been demonstrated in mammalian exosomes, providing new exploring opportunities for plant-derived EVs, for the research community. Only a few drug delivery systems, based on plant-derived extracellular vesicles, have entered clinical trials and although preliminary findings advocate a new, exciting era for therapeutic nanocarriers, many challenges remain until they mature into clinical translation. Among these are, the standardization of separation techniques suitable for mass production and their efficient purification, with the selection of the most appropriate EVs for a specific use, being arguably the greatest challenge of all.

**Table 1:** Plants used for the extraction of plant-derived EVs.

Common Name	FAO ^1^ Group	Scientific Name	Sample	Isolation Method	Ref.
Peas	4—PULSES AND DERIVED PRODUCTS	*Pisum sativum*	Seed juice	Differential centrifugation/filtration/ultracentrifugation	[20]
Sunflower seeds	6—OIL-BEARING CROPS AND DERIVED PRODUCTS	*Helianthus annuus*	Seeds extracellular fluids	Vacuum infiltration/centrifugation procedure/differential centrifugation/ultracentrifugation	[24]
Coconut		*Cocos nucifera* L.	Coconut water or coconut milk	Differential centrifugation/filtration/ultracentrifugation	[20,23]
			Coconut juice		
Soybean		*Glycine soja*	Bean juice		
Carrot	7—VEGETABLES AND DERIVED PRODUCTS	*Daucus carota*	Root juice	Density gradient centrifugation	[13,29]
Broccoli		*Brassica oleracea*	Flowering head and stalk juice		
Tomatoes		*Lycopersicon esculentum*	Tomato juice	Density gradient centrifugation Differential centrifugation/filtration/ultracentrifugation	[5,18]
Watermelons		*Citrullus lanatus*	Mesocarp juice	Differential centrifugation/filtration/ultracentrifugation	[18,27]
Melons		*Cucumis melo*	Fruit juice		
Garlic		*Allium sativum*	Clove juice	Density gradient centrifugation	[29]
				Differential centrifugation/filtration/ultracentrifugation or density gradient centrifugation	[12]
Grapes	8—FRUITS AND DERIVED PRODUCTS	*Vitis vinifera*	Fruit juice	Density gradient centrifugation	[8,9,17]
				Differential centrifugation/filtration/ultracentrifugation	[18]
Lemons		*Citrus limon* L.	Fruit juice	Density gradient centrifugation	[22]
				Differential centrifugation/filtration/ultracentrifugation	[5]
Grapefruit		*Citrus paradisi*	Fruit juice	Density gradient centrifugation Lipid re-assembling via sonication Differential centrifugation/filtration/ultracentrifugation or density gradient centrifugation	[7,12,15,17,19,20,29,34]
Blueberries		*Vaccinium myrtillus* *Vaccinium corymbosum*	Fruit juice	Differential centrifugation/filtration/ultracentrifugation	[20]
Kiwis		*Actinidia chinensis*			
Oranges		*Citrus sinensis*			
Pears		*Pyrus communis*			
Pineapples		*Ananas comosus*	Fruit juice	-	[42]
Ginger	10—SPICES	*Zingiber officinale*	Root Juice	Density gradient centrifugation Lipid re-assembling via film hydration/sonication/extrusion method Differential centrifugation/filtration/ultracentrifugation or density gradient centrifugation	[9,11,12,19,20,21,25,28,29]
Turmeric		*Curcuma longa*	Root Juice	Density gradient centrifugation Differential centrifugation/filtration/ultracentrifugation or density gradient centrifugation	[12,29]
Cilantro (coriander)		*Coriandrum sativum*	Leaf juice	Differential centrifugation/filtration/ultracentrifugation or density gradient centrifugation	[12]
Aloe vera	-	*Aloe vera barbadensis*	Leaf juice	Differential centrifugation/filtration/ultracentrifugation or density gradient centrifugation	[12]
Dandelion		*Taraxacum*			
Lavender		*Lavandula*			
Cactus		*Cactus*	Stem juice	Differential centrifugation/filtration/ultracentrifugation or density gradient centrifugation	[12]
Tobacco leaves	13—TOBACCO AND RUBBER AND OTHER CROPS	*Nicotiana tabacum*	Apoplastic fluid from fresh or dried leaves	Vacuum infiltration/centrifugation/differential centrifugation	[27]
Lesser periwinkle plant	-	*Vinca minor* L.	Apoplastic fluid from fresh or dried leaves	Vacuum infiltration/centrifugation/differential centrifugation	[27,31]
European mistletoe plant		*Viscum album* L.			
Arabidopsis plant		*Arabidopsis thaliana*			

^1^ Food and Agriculture Organization.

**Table 2:** Physicochemical characterization of plant-derived EVs.

Plant	Size (nm)	Morphology	z-Potential (mV)	Delivery Route	Effect/Disease	Therapeutic Biomolecule	Loading (%) Enc. Eff. (%) ^9^	Ref.
Grapes	200–800 (EM ^1^, DLS ^2^) 380 (av. diam. ^3^)	Spherical	~−27	Oral	DSS-induced colitis ^4^	-	-	[8]
	500–1000 (EM ^1^, DLS ^2^)	Spherical or cup-shaped	~−40		Intestinal homeo-stasis	-	-	[9]
	30–200 (EM ^1^)	Spherical	-	-	-	-	-	[18]
Grape-fruits	50–800 (EM ^1^, DLS ^2^) 253 (av. diam. ^3^) *Re-ssembled GrfNVs* ^5^ 50–400 (EM ^1^, DLS ^2^) 180 (av. diam. ^3^)	Spherical or cup-shaped *Re-assembled GrfNVs* ^5^ Flower-like	-	i.v. ^6^ injection	Cancer	PTX ^7^ Folic acid	-	[17]
	50–100 100–1000 (EM ^1^, DLS ^2^)	Spherical or cup-shaped	~−40	Oral	Intestinal homeo-stasis	-	-	[9]
	105–400 (EM ^1^, DLS ^2^) 210 (av. diam. ^3^)		~−25		DSS-induced colitis ^4^	Methotrexate	-	[7]
	*GrfNVs*^5^ ~200 *IGrfNVs*^8^ ~200 (EM ^1^)	*GrfNVs*^5^ spherical *IGrfNVs* ^8^ spherical	*GrfNVs*^5^ negative *IGrfNVs* ^8^ negative	i.v. ^6^ injection	DSS-induced colitis ^4^ Inflammation	Doxorubicin Curcumin	*Enc. Eff. (%)*^9^ *Dox-IGrfNVs*^10^ 70–80 *Cur-IGrfNVs*^11^ 50–60	[15]
	*GrfNVs*^5^ 110 (av. diam. ^3^) *OGrfNVs*^12^ 120 (av. diam. ^3^) (DLS ^2^)	-	-		Liver metastasis	miR-18a	-	[34]
	*GrfNVs*^5^ 102.4 (av. diam. ^3^) *pGrfNVs*^13^ 87.2 (av. diam. ^3^) *FA-pGrfNVs*^14^ 72.4 (av. diam. ^3^) (DLS ^2^)	Spherical	*GrfNVs*^5^ −38.15 *pGrfNVs*^13^ −13.9 *FA-pGrfNVs*^14^ −31	Intranasal	Brain tumor	miR17	*Enc. Eff. (%)*^9^ *GNVs*^5^ <20 *pGNVs*^13^ 80–90	[19]
	2 populations (DLS ^2^)	Spherical or oval (AFM ^15^)	-	-	-	-	-	[20]
Ginger	100–1000 (EM ^1^, DLS ^2^)	Spherical or cup-shaped	~−25	Oral	Intestinal homeostasis	-	-	[9]
	*GiNVs*^16^ 100–1000 (DLS ^2^) 386 (av. diam. ^3^) *GiNVs2*^17^ 100–1000 (DLS ^2^) 294 (av. diam. ^3^)	Spherical (AFM ^15^)	*GiNVs*^16^ −24.6 *GiNVs2*^17^ −29.7	Oral	Liver-related diseases	-	-	[10]
	*Re-assembled GiNVs*^18^ 188 (av. diam. ^3^) (DLS ^2^) *Dox-GiNVs*^19^ 188 (av. diam. ^3^) (DLS ^2^)	Spherical or cup-shaped *Re-assembled GiNVs* ^18^Spherical *Dox-GiNVs* ^19^Spherical	~−16	i.v. ^6^ injection	Colon cancer	Doxorubicin	*Dox-GiNVs*^19^ Up to 95.9% ± 0.26%	[28]
	*GiNVs_1*^18^ 292 (av. diam. ^3^) (DLS ^2^) *GiNVs_2*^18^ 231 (av. diam. ^3^) (DLS ^2^) *GiNVs_3*^18^ 219 (av. diam. ^3^) (DLS ^2^)	Spherical or cup-shaped	(−12.9)–(−2.1)	Oral	Inflammatory bowel disease Colitis-associated cancer	-	-	[25]
	*GiNVs*^16^ 232 (av. diam. ^3^) *Reassembled GiNVs*^16^ 189 (av. diam. ^3^) (EM^1^, DLS ^2^)	Spherical	~−18	Oral	Ulcerative colitis	siRNA-CD98	-	[11]
	2 populations (DLS ^2^)	Spherical or oval (AFM ^15^)	-	-	-	-	-	[20]
	50–150 (EM ^1^)	Spherical	-	Oral	Gut diseases		-	[29]
	120–150 (EM^1^, NTA ^21^)		-		inhibit NLRP3 inflammasome activity ^20^ (Alzheimer’s disease)	-	-	[12]
	100–600 (NTA ^21^)	-	-	Oral cavity	Periodontitis	-	-	[21]
Carrots	80–200 and 700–1500 (EM ^1^, DLS ^2^)	Spherical or cup-shaped	~−25	Oral	Intestinal homeostasis	-	-	[9]
Lemons	50–70 (EM ^1^, DLS ^2^)	Spherical	-	Intra tumor (locally) Intraperitoneally	Cancer	-	-	[22]
	30–100 (EM^1^)	Spherical or cup-shaped	-	-	-	-	-	[5]
Broccoli	18–120 32 (av. diam. ^3^) (EM ^1^, DLS ^2^)	Spherical	−17	Oral	Colitis	-	-	[13]
Sun-flower seeds	50–200 (EM ^1^)	Spherical (phospho-lipid layer)	-	-	-	-	-	[24]
Coconut	*EVs from coconut water* 13 (EM ^1^) and 60 (DLS ^2^) *EVs from coconut milk* 30 (EM ^1^) and 100 (DLS ^2^)	Spherical	-	-	-	-	-	[23]
	2 populations (DLS ^2^)	Spherical or oval (AFM ^15^)	-	-	-	-	-	[20]
Water-melons	100–200 (EM ^1^) 32 (av. diam. ^3^) (NTA ^21^)	Cup-shaped	-	-	-	-	-	[30]
Blue-berries	2 populations (DLS^2^)	Spherical or oval (AFM ^15^)	-	-	-	-	-	[20]
Kiwis	>2 populations (DLS ^2^)		-	-	-	-	-	
Oranges	2 populations (DLS ^2^)		-	-	-	-	-	
Peas	2 populations (DLS ^2^)		-	-	-	-	-	
Pears	2 populations (DLS ^2^)		-	-	-	-	-	
Soybean	2 populations (DLS ^2^)		-	-	-	-	-	
Melons	40–70 and 100–1000 (DLS ^2^)		-	-	-	-	-	
Tomatoes	100–1000 (DLS ^2^)		-	-	-	-	-	
Ara-bidopsis leaves	*P40 fraction*^22^ 50–300 150 (av. diam. ^3^) (EM^1^, DLS ^2^) *P100 fraction*^22^ 10–17 12 (av. diam. ^3^) (EM ^1^, DLS ^2^)	spherical	-	-	-	-	-	[31]
Tobacco leaves	70 ± 20 and 520 ± 170 (EM ^1^)		-	-	-	-	-	[27]
Lesser peri-winkle plant	380 ± 200 (EM ^1^)		-	-	-	-	-	
European mistletoe plant	280 ± 115 (EM ^1^)		-	-	-	-	-	

^1^ Electron Microscopy; ^2^ Dynamic Light Scattering; ^3^ Average diameter; ^4^ Dextran sulfate sodium-induced colitis; ^5^ Grapefruit-derived nanovesicles; ^6^ Intravenous; ^7^ Paclitaxel; ^8^ Coated with inflammatory related receptor enriched membranes of activated leukocytes; ^9^ Encapsulation efficiency; ^10^ Doxurubicin-loaded; ^11^ Curcumin-loaded; ^12^ Optimized GrfNVs; ^13^ made of PEI/RNA and GrfNV complex; ^14^ Folic acid pGrfNVs; ^15^ Atomic Force Microscopy; ^16^ Ginger-derived nanovesicles from band 1 of the sucrose gradient; ^17^ Ginger-derived nanovesicles from band 2 of the sucrose gradient; ^18^ Ginger-derived nanovesicles; ^19^ Doxurubicin-loaded ginger-derived nanovesicles; ^20^ pyrin domain-containing 3 (NLRP3) inflammasome is a key regulator of innate immune responses, and its activation is implicated in the pathogenesis of many diseases, such as Alzheimer’s disease and type 2 diabetes; ^21^ Nanoparticle Tracking Analysis; ^22^ 40,000× and 100,000× *g* in centrifugation.

**Table 3:** Lipidomic analysis of plant-derived vesicles.

Plant	Prevalent Bioactive Lipids	Ref.
Grapes	PA ^1^ (53%) PE ^2^ (26%)	[8]
Grapefruits	DGDG ^4^ (24%) PC ^3^ (23%) DAG ^6^ (17%) MGDG ^5^ (~13%)	[17]
	PE ^2^ (46%) PC ^3^ (29%)	[7]
	PC ^3^ (36%) PE ^2^ PA ^1^ (3.5%)	[29]
Ginger	PA ^1^ (37–40%) DGDG ^4^ (33–40%) MGMG ^8^ (17–20%)	[10]
	PA ^1^ (48%) MGDG ^5^ (28%) DGDG ^6^ (15%)	[28]
	PA ^1^ (~25–40%) DGDG ^4^ (~25–40%) MGDG ^5^ (~20–30%)	[25]
	PA ^1^ (42%) DGDG ^4^ (27%) MGDG ^5^ (19%).	[11]
	PA ^1^ (35%) MGMG ^8^ DGDG ^4^	[29]
	PC ^3^ (48%) TAG ^9^ (9%)	[21]
Turmeric	PA ^1^ (34%) MGMG ^8^ DGDG ^4^	[29]
Sunflower seeds	PA ^1^ PI ^7^	[24]
Garlic	PC ^3^ (53%) PE ^2^ PA ^1^ (5.5%)	[29]

^1^ Phosphatidic acids; ^2^ Phosphatidylethanolamines; ^3^ Phosphatidylcholine; ^4^ Digalactosyldiacylglycerol; ^5^ Monogalactosyldiacyglycerol; ^6^ Diacylglycerol; ^7^ Phosphatidylinositol; ^8^ Monogalactosyl monoacylglycerol; ^9^ Triacylglycerol.

**Table 4:** Plant-derived EVs in clinical trials.

Condition/Disease	Year/Phase	EV Source	Administration	Therapeutic Molecule	Results/Status	Ref.
Colon cancer (NCT01294072)	2011 Phase I Clinical Trial	Plants	Tablets oral, daily for 7 days	Curcumin	Active, not recruiting	[37]
Oral Mucositis (NCT01668849)	2012 Phase I Clinical Trial	Grapes	Dietary Supplement oral, daily for 35 days	-	Active, not recruiting	[43]
Insulin-related conditions Chronic inflammation in POS ^1^ patients (NCT03493984)	2018 Preliminary Clinical Trial	Ginger Aloe	-	-	Recruiting	[38]

^1^ Polycystic ovary syndrome.
